# The Emergence of Creative Leaders Within Social Networks: The Case of Andy Warhol in the Art World of New York

**DOI:** 10.3389/fpsyg.2021.635678

**Published:** 2021-07-05

**Authors:** Marios Samdanis, Soo Hee Lee

**Affiliations:** ^1^Brunel Business School, Brunel University London, Uxbridge, United Kingdom; ^2^Kent Business School, University of Kent, Canterbury, United Kingdom

**Keywords:** creative leader emergence, art world, achievement, ascription, social networks, atypicality

## Abstract

The creative leadership literature has identified personality traits, skills, states, and behaviours which are effective within creative contexts and organisations, but it is yet to address how creative leaders emerge from social networks. This conceptual paper delineates the processes of creative leader emergence within the context of contemporary visual arts. Using a relational view of creative leader emergence, this paper incorporates the leader emergence processes of achievement and ascription, and then adjusts them to the context of the art world. We argue that both competence and identity contribute to the status construction of creative leaders by enabling their emergence within social networks. In addition to the processes of leader prototypicality through which leaders emerge within groups, we also identify processes of leader atypicality through which creative leaders emerge within network structures. Finally, our conceptual analysis is illustrated by the case of Pop artist Andy Warhol, focusing on his emergence as a creative leader within the art world of New York and his art studio, the factory.

## Introduction

The literature on creative leadership highlights personality traits, skills, states, behaviours, and styles of effective creative leaders (Mainemelis et al., 2015; Epitropaki et al., 2018), but it does not address how creative leaders emerge. The term leader emergence refers to “whether (or to what degree) an individual is viewed as a leader by others” (Judge et al., 2002, p. 67). Leader emergence and creativity are similar constructs, as they are both rely on the perceptions and beliefs of others (Epitropaki et al., 2018): Leader emergence is endorsed by followers (Lee and Farh, 2019), and creative outcomes are recognised by experts and audiences (Bourdieu, 1993).

This paper provides a conceptual analysis of creative leader emergence in the context of contemporary visual arts, responding to recent calls in the literature on creative leadership for context-specific investigations of individual creative leaders (Epitropaki et al., 2018; Randel and Jaussi, 2019). This research aims to address how creative leaders emerge in social networks. The creative leadership literature mainly scrutinises the role of traits, perceptions, skills, behaviours and creative contexts in leadership effectiveness within groups, networks and organisations (Mumford et al., 2003; Mainemelis et al., 2015; Abecassis-Moedas and Gilson, 2018; Epitropaki et al., 2018). Creative leadership emergence has attracted interest recently, as Randel and Jaussi (2019) identify its contextual enablers and redundancies within organisations. In this paper, we aim to complement current research by shifting the context of analysis from organisations to social networks, focusing explicitly on the processes of creative leader emergence. Artists emerge as creative leaders from social networks or art worlds (Becker, 1982). According to Becker (1982), the creation of art is a collective action that takes place in an art world, which consists of a social network of interrelated agents, including artists, their assistants, peers, experts, and audiences. Although art is created collectively, artists have a leading role in this process (Becker, 1982), which we aim to explore in this paper.

To address how creative leaders emerge from social networks, we synthesise the literature on leader emergence (Paunova, 2015) and the sociology of art (Becker, 1982; Bourdieu, 1993). Creative leader emergence is conceptualised as an emergent, multilevel and relational process, which is determined by the interactions of creative leaders and others (e.g., followers, peers, partners, experts) within social networks (Uhl-Bien et al., 2014; Acton et al., 2019). These interactions produce perceptions, status, beliefs, identities and discourses that shape the meaning of emergence within a social context (Uhl-Bien, 2006; Paunova, 2015; Oc, 2018).

Paunova (2015) identifies two leader emergence processes, achievement and ascription, which are incorporated in our analysis and adjusted to the context of the art world (Becker, 1982). Both processes are based on the premise that leaders emerge because followers acknowledge their status, which is defined as “the respect, admiration, and voluntary deference individuals are afforded by others” (Anderson et al., 2015, p. 574). Achievement is the process of emergence through which a leader's status is endorsed by followers, due to their competence, functional behaviours and differentiation (DeRue et al., 2015; Paunova, 2015). Ascription is the process of leader emergence through which a leader's status derives from their nominal characteristics and social identities, such as gender, race, class or sexual identity (Paunova, 2015). We claim that both processes are required for the emergence of a creative leader; however, the two processes need adjustment before they can be applied to the context of the art world.

The achievement mechanism can only partly explain the emergence of creative leaders in the art world. Leader emergence has mostly been studied within existing groups (Judge et al., 2002; DeRue et al., 2015; Paunova, 2015) or leaderless groups (Ensari et al., 2011; Lee and Farh, 2019). In this research, emergence, is addressed in terms of leader prototypicality, as a leader's traits, states, skills and behaviours match the image of them that their followers have in their minds (Epitropaki et al., 2018). In the visual arts, achievement for creative leaders often refers to receiving recognition for their deviations from aesthetic conventions (Stamkou et al., 2018). However, creative leaders do not necessarily emerge within existing groups, but within networks in the art world which consist of peers, experts and audiences who endorse their artistic deviance (Becker, 1982). Current views, such as that of Paunova (2015), according to which leaders emerge because they can behave in ways that better serve the needs of a group; or because their traits match the image that their followers have in their minds, are not sufficient to explain creative leader emergence.

The emergence of creative leaders depends on their competence in a creative field, but also on their social identities (Mainemelis et al., 2015). Ascription processes focus on the role played by the social identities of leaders in constructing their status (Paunova, 2015). Ascription processes alone can lead to the reproduction of stereotypes that associate higher status with particular demographics, such as white, male, heterosexual or upper-class leaders in the Western context (Samdanis and Özbilgin, 2020). The ascription process can have a different meaning in the art world, as artists often develop atypical identities that diverge to varying degrees from established norms within a social context. Once these identities are endorsed by their followers, creative leaders can enact a creative context, as new cooperative links emerge based on a shared lifestyle, ideology or friendship (Becker, 1982; Kwok et al., 2018).

Overall, we argue that both competence and identity contribute to the status construction of creative leaders, and that they are essential enablers for their emergence within social networks. In this paper, our conceptual analysis is illustrated by a historical case study of Andy Warhol's emergence as a creative leader in his art organisation the Factory, and as a leading figure in the American Pop Art movement in the 1960s. Warhol received significant recognition in New York's art world, nurtured an underground artistic milieu at the Factory, and manifested his atypical identity through the construction of his artistic persona (Bockris, 2003; Gopnik, 2020).

## The Emergence of Creative Leaders

The creative leader is the primary source of creative thinking and behaviour within a creative context, aiming to lead others to the attainment of a creative outcome (Mainemelis et al., 2015). Creative leaders are conceptualised as effectively managing a group or team throughout a creative process, being involved at the stages of idea generation, structuring, implementation and promotion (Conger, 1995; Mumford et al., 2003; Oliver and Ashley, 2012; Mainemelis et al., 2015; Lee and Farh, 2019). Creative leaders are often portrayed as charismatic, possessing a unique set of personality traits, as well as creative problem-solving and technical skills that enable them to manage emergent, complex and multilevel processes of creativity and innovation within organisations (Anderson et al., 2014; Epitropaki et al., 2018).

However, those personality traits that enable leader emergence diverge from the traits of successful creative individuals. By reviewing implicit theories of leadership and creativity^1^, Epitropaki et al. (2018) compare the traits and attributes of leader prototypicality (i.e., the degree to which a leader's traits match the image their followers have in their minds) with the traits and attributes of creative individuals. Traits such as being intelligent, honest, understanding, determined and decisive are prototypical in leaders, but differ significantly from the traits of creative individuals, which include being open-minded, open to new experiences, imaginative, intelligent, curious and resourceful. Apart from intelligence, which is a common trait, participants seem to appreciate different traits and attributes in leaders and creative individuals. Epitropaki et al. (2013, p. 873) point out that in implicit leadership theory (Lord et al., 1984) “the trait ‘creative' was included in the non-leader attributes list which clearly indicates that creativity is not viewed as a core characteristic of leadership.”

Creative leaders are appreciated by others both within and beyond an organisational setting for their ability to respond to dynamic, innovation-driven environments (Conger, 1995; Anderson et al., 2014; Randel and Jaussi, 2019). As a result, the personality traits that followers appreciate in their creative leaders may vary according to the creative environment. As Epitropaki et al. (2018) claim, the traits of creative individuals emerge as salient in the minds of followers on the fly, as influenced by situated leader-follower interactions, task characteristics and the context of creativity.

The current literature on creative leadership situates the study of creative leaders within creative contexts (Mainemelis et al., 2015, 2018). One of the most influential studies conducted by Mainemelis et al. (2015) introduces a multi-context framework of creative leadership. This framework identifies the creative-leadership contexts of directing, facilitating and integrating, based on the extent to which creative leaders combine their creative contributions with those of their followers. More specifically, directing is the context in which creative leaders are the main source of creative contributions, while the work of followers is restricted to materialising the creative visions of leaders; in the facilitating context, followers are the main source of creative contributions, and creative leaders foster the creativity of followers; and in the integrating context, a creative outcome relies on the creative contributions of both leaders and followers, and thus creative leaders aim to synthesise heterogeneous creative work and inputs (Mainemelis et al., 2015).

The multi-context framework of creative leadership has been widely applied across various creative fields, including choreography (Abecassis-Moedas and Gilson, 2018), filmmaking (Flocco et al., 2018), TV production (Dovey et al., 2017), and haute cuisine (Mainemelis et al., 2015). For instance, in managing the creation of advertising, creative leaders are often responsible for attracting highly motivated, curious, and creative individuals, while also creating an environment which nurtures innovation by being fun, energy-charged and supportive of risk-taking (Oliver and Ashley, 2012). In this context, the creative leader facilitates the creativity of others by finding new and improved ways of working, managing conflict within and across teams, and preventing the burnout of creative individuals (Oliver and Ashley, 2012; Anderson et al., 2014).

In addition, Abecassis-Moedas and Gilson (2018) argue that within collective artistic contexts, leaders are usually creatives, and are thus more inclined to behave in a directive and integrative way, and less in a facilitative way. This is likely to happen because the “identity of the leader is often closely tied to the outcome” (Abecassis-Moedas and Gilson, 2018, p. 125). Based on 20 case studies of choreographers, the authors found that creative leaders employ directive or integrative behaviour, depending on the nature of each project, their general perspective on the role of followers and their personal preferences (Abecassis-Moedas and Gilson, 2018). An important finding of this research, consistent with prior research on creativity and leadership (Mumford et al., 2003; Anderson et al., 2014), is that effective creative leaders should provide both autonomy and guidance to followers (Abecassis-Moedas and Gilson, 2018). In filmmaking, creative production relies on integrating heterogeneous skills and labour, while film directors as creative leaders are responsible for integrating diverse inputs and work into the final cut (Flocco et al., 2018).

The multi-context framework (Mainemelis et al., 2015) is a powerful analytical tool used for identifying patterns of creative-leader behaviour based on the characteristics of creative tasks and the physical, social, organisational, industrial and temporal contexts in which creative leaders operate (Mainemelis, 2018; Oc, 2018). However, this approach does not fully or directly address the ways in which creative leaders emerge. Mainemelis (2018) later argued that leadership behaviours that are considered prototypical are all constrained by their contexts. This view explains the emergence of creative leaders, based on their competence and functional behaviours, which are perceived as prototypical within a creative context.

However, the emergence of leaders may depend not only on their functional behaviours within a particular context, but also on their social identities, which are perceived as valuable by their followers (Paunova, 2015). Paunova (2015) delineates two enactment mechanisms for leader emergence: achievement and ascription. The achievement mechanism explains how leaders emerge based on their functional behaviours, which are perceived as the antecedents of leadership perceptions within a group (“i.e., trait → state → behaviour → perception”) (Paunova, 2015, p. 938). Mental states, such as an individual's self-motivation (Paunova, 2015), creative self-efficacy (Huang et al., 2016), creative problem-solving and social skills (Mumford et al., 2003), derive from personality traits, and influence behaviours, which are perceived and evaluated by others to be leader-like. Sirén et al. (2020) studied leadership in nascent venture teams, and found that trait dispositions, such as regulating emotion, have a positive effect on leader emergence. The quality of talk or speaking up promotively are both associated with higher peer-rated status, which enables leader emergence (Paunova, 2015; McClean et al., 2018). The behaviours of leaders are functional once they are required for a group to function properly, and a member of a group who displays functional behaviour is likely to be rewarded with higher leadership status (Paunova, 2015). Certain creative-leader behaviours support the emergence of creative leaders because they show the followers that those individuals are both competent and a good fit with the purposes of the group.

The ascription mechanism emphasises the importance of traits and nominal demographics, such as gender or race, as antecedents of leadership perceptions (“i.e., trait → perception → state → behaviour”) (Paunova, 2015:938). According to the ascription mechanism, leaders emerge as their observable characteristics and social identities (e.g., gender, race, class) shape the perceptions of their followers (Paunova, 2015; Samdanis and Özbilgin, 2020). Key to the ascription mechanism is the concept of status beliefs, which lead people to “associate greater status and general competence with people in one social category than another, while granting those in each category some specialised skills” (Ridgeway, 2011, p. 60). In addition to the social identities of leaders, their appearance, sexual orientation, self-efficacy, and style also influence the perceptions of followers. The ascription mechanism relies on implicit theories of leadership and creativity, because certain traits, such as being insightful, quirky, disobedient or impulsive, are perceived by followers as those of creative individuals (Epitropaki et al., 2018).

Based on Paunova (2015), we develop the achievement- and ascription-based pathways to explain the emergence of creative leaders. The achievement pathway is usually based on in-group processes of leader prototypicality. Prototypical leaders do not only comply with the norms and expectations of a group, but also demonstrate and sustain their differentiation and competence over time (Halevy et al., 2011; DeRue et al., 2015). This process of creative leader emergence is rooted in the idiosyncrasy credit theory, according to which leaders emerge by first exhibiting competence and conformity to a group to accumulate credit, then redeeming this credit by deviating from established practises (Hollander, 1958). The ascription pathway offers an alternative process for creative leader emergence, based on atypicality instead of prototypicality (Samdanis and Özbilgin, 2020). Atypicality is the degree to which a leader's social identity deviates from those of typical and prototypical leaders within a social context. Leaders may emerge by manifesting their atypical identities through discourses, dispositions and performative acts, while followers may endorse a creative leader because they subscribe to similar values, styles, lifestyles and ideologies, which are interwoven with the atypical identity of the creative leader. As a result, a network of followers can be created, as they endorse the identity of a creative leader in addition to their competence.

## The Emergence of Creative Leaders in the Art World: A Conceptual Refinement of Achievement and Ascription

Current research mainly regards creative leadership as an in-group organisational phenomenon (Mumford et al., 2003; Abecassis-Moedas and Gilson, 2018; Epitropaki et al., 2018; Randel and Jaussi, 2019). Research within the sociology of art suggests that cultural production and consumption take place within social networks (Becker, 1982; Bourdieu, 1993; Jones, 2010). The emergence of creative leaders in contemporary visual arts is situated within the context of the art world. According to Becker (1982), the creation of art does not result from the work of an isolated creative genius, but is the outcome of collective action that takes place within an art world. Becker defines the term art world as a “network of people whose cooperative activity” is “organised via their joint knowledge of conventional means of doing things,” producing “the kind of artworks that the art world is noted for” (Becker, 1982, p. X). An art world is a social organisation or a creative network of interrelated agents, including artists and their assistants, art dealers, critics, curators, collectors and the audience that share similar tastes and aesthetic conventions (Becker, 1982).

The creation of art as a collective action requires a certain division of labour within the art world, as well as processes of coordination through which heterogeneous resources are integrated (Becker, 1982; Mainemelis et al., 2015). Its conventions rely on the knowledge, expertise and experience of artists, experts and audiences, and facilitate the appreciation of art as shared mental representations of taste and aesthetics, which emerge in relation to the conceptual, stylistic and technical aspects of an artwork. These conventions are cognitive mechanisms that coordinate agents in the art world, once creative individuals have emerged as leaders (Bastardoz and Van Vugt, 2019). However, Becker (1982) does not make it clear whether such conventions are appreciated by followers solely on the basis of artistic merit, supporting the achievement process of creative leader emergence, or whether they also refer to the social identities shared by leaders and followers in the art world, a view aligned with the ascription process of creative leader emergence.

In the following sections, we extend the creative leader emergence processes of achievement and ascription, adjusting them to the context of the art world. Artistic deviance and recognition are identified as key enablers for achievement-based processes of creative leader emergence; while networking and atypicality are identified as key enablers for ascription-based processes of creative leader emergence.

### Achievement-Based Processes of Creative Leader Emergence

#### Artistic Deviance

The emergence of creative leaders in contemporary visual arts relies on processes through which creative individuals gain status, recognition as artistic innovators and followers in the art world (Stamkou et al., 2018; Svejenova, 2018). Creative outcomes are considered novel when artists deviate from “their own previous style (intrapersonal deviance) and other artists' styles (interpersonal deviance)” and “deviance is directed toward a progressive style” (Stamkou et al., 2018, p. 276). More broadly, the term social deviance describes a “phenomenon which is perceived… as violating expectations held by participants in an event” (Hawkins and Tiedeman, 1975, p. 59), often referring to criminal behaviour when “individuals fall below the legally prescribed norms of moral conduct” (Sorokin, 1950, p. 81). However, social deviance can also refer to “variations from social norms in desirable and enviable directions” (Lemert, 1951, p. 23–24), which are observed in the cases of outstanding athletes, scientists or artists (Heckert, 1989). In this paper, we focus on artistic deviance as an act of violating established aesthetic norms and conventions (Stamkou et al., 2018). Heckert (1989) notes that artists such as the Impressionists in Paris were initially designated as negative deviants by the artistic establishment and received criticism for violating aesthetic conventions, but later, once they received recognition, their status elevated to positive deviance.

Artistic deviance and convention appear to be contrasting terms. On the one hand, conventions enable artists to gain access to resources, but producing work that is too conventional may result in few rewards (Becker, 1982). On the other hand, when individuals deviate too much too early, this is unlikely to receive the support of others which is necessary for their emergence as leaders (Stone and Cooper, 2009). This paradox was identified by Hollander (1958), who tried to explain how leaders emerge in groups: “One must conform to group norms in order to be accepted as a member of a group, but one has to deviate from group norms to lead the group” (Stone and Cooper, 2009, p. 787). This is the premise of Hollander's (1958) idiosyncrasy credit theory, which states that leaders emerge by first exhibiting competence and conformity to a group in order to accumulate credit, and then redeem their credit by deviating from established practises (Hollander, 1958, xbib1960). The achievement approach to leader emergence supports the theory that creative leaders build their status, esteem and influence on the foundation of their perceived competence (Stone and Cooper, 2009; DeRue et al., 2015).

The emergence of creative leaders in the contemporary art world is due to their competence in introducing new conventions and sustaining their progressive style over time (Becker, 1982; Stamkou et al., 2018). The ultimate acceptance of new conventions also legitimises the context and behaviour of creative leaders (Mainemelis et al., 2015). Such an achievement-based mechanism explains how creative leaders emerge over time by first building their status and then deviating. However, this transactional approach to creative leader emergence should address not only how a creative leader emerges, but also why others acknowledge the new conventions introduced by him or her.

#### Recognition

Creative leaders emerge within social networks, when their aesthetic conventions receive recognition from powerful intermediaries and experts (Bourdieu, 1993; Delacour and Leca, 2017). Although artists create new conventions in the art world, they cannot themselves make legitimate claims about the aesthetic novelty and value of their art. Instead, their achievement must be acknowledged by experts and intermediaries who use their power, position, knowledge and recognition (their *cultural, symbolic, and social capital*) to consecrate and promote legitimately distinctive art (Bourdieu, 1984). Intermediaries can also be creative leaders. In the field of music production, producers act as creative leaders and brokers, connecting previously dispersed artists, artists' managers and record labels (Lingo and O'Mahony, 2010; Lingo, 2018). Art dealers can act as creative leaders and brokers, introducing the work of artists to curators or art collectors (Bourdieu, 1993).

Artists emerge as creative leaders once experts within social networks have legitimised their status and justified their competence (Cattani et al., 2015). Firstly, experts play an important role in the emergence of creative leaders, as they justify the cultural importance of their work, using discourses; while, in some cases, they can even construct the identity of artists, labelling their work (Bourdieu, 1984). Secondly, processes of art legitimisation may result in a shift in the position of creative leaders from the periphery of an art world (i.e., an avant-garde position) into the mainstream (Alvarez et al., 2005; Patriotta and Hirsch, 2016). This shift in the position-taking of a creative leader can also be seen as a process of emergence, based on the recognition of their conventions by experts and broader audiences.

### Ascription-Based Processes of Creative Leader Emergence

#### Networking

The ascription approach to creative leader emergence focuses on the role of leaders' observable characteristics (e.g., age, appearances, and styles) and social identities (e.g., gender, race, sexual orientation, and class) as antecedents of leadership perceptions (Paunova, 2015). The ascription mechanism emphasises the role of affection and social identity—in addition to competence—in the emergence of creative leaders (DeRue et al., 2015; Paunova, 2015). Put simply, creative leaders may network with others in the art world not just because they create innovative, progressive work (Stamkou et al., 2018), but also because of their personality traits, such as warmth, benevolence, likeability, and trustworthiness (DeRue et al., 2015). Traits, such as being friendly and approachable, are identified in the leadership literature in explaining how leaders emerge by gaining the support of followers through building friendship ties, kinship and acquaintance (Uzzi and Gillespie, 2002; Kwok et al., 2018).

Creative leaders also emerge in the art world as a result of their ascription, as their social identities are perceived as creative-like by others (Epitropaki et al., 2018). Firstly, the definition of conventions in this ascriptive approach is broader than that of aesthetic conventions, as it includes social identities, appearances, discourses and styles which can be shared between creative leaders and others within the art world. Becker (1963) in his earlier work analysed the language of jazz musicians, claiming that their use of slang unites a group and differentiates it from others (Cluley, 2012). Secondly, unlike the achievement processes of creative leader emergence, in which the status of leaders is validated by an existing art world, ascriptive processes are likely to enact a new art world based on identity work as “individuals co-create reciprocal and mutually reinforcing identities as leaders and followers and, through this process, develop a leader-follower relationship” (DeRue and Ashford, 2010, p. 628).

#### Atypicality

In ascription processes, creative leaders emerge by consciously engaging with self-stylisation, self-exposure and self-display to influence the perceptions of others (Finkelstein, 2007). A new mode of artist-personality emerges by blending art, image and celebrity (Stallabrass, 2006). This form of ascription is based on the assumption that artists can actively construct their identities, which in turn can attract endorsement from others. This cultural tactic is known as the creation of an artistic persona, defined as “a trajectory of contemporary art in the post-industrial art world in which artists' activities increasingly include non-art services such as networking and mass media publicity” (Lee, 2015, p. 27).

The artist duo Gilbert and George is known for their formal appearance and manner, as persona is “all-important, and inseparable from the art in which they appear” (Stallabrass, 2006, p. 47). The feminist artist Tracey Emin has engaged with self-exposure, using “her art for a process of transferring her life and memories into a public artwork” (Remes, 2009, p. 560). Having its conceptual origins in Goffman's (1959) dramaturgical framework and Jung's (1953) persona, artists, and creative leaders can manipulate their public image through discourses, dispositions and performances to construct an atypical identity to influence the perceptions of others (Fawkes, 2015; Samdanis and Özbilgin, 2020).

**Figure 1:**
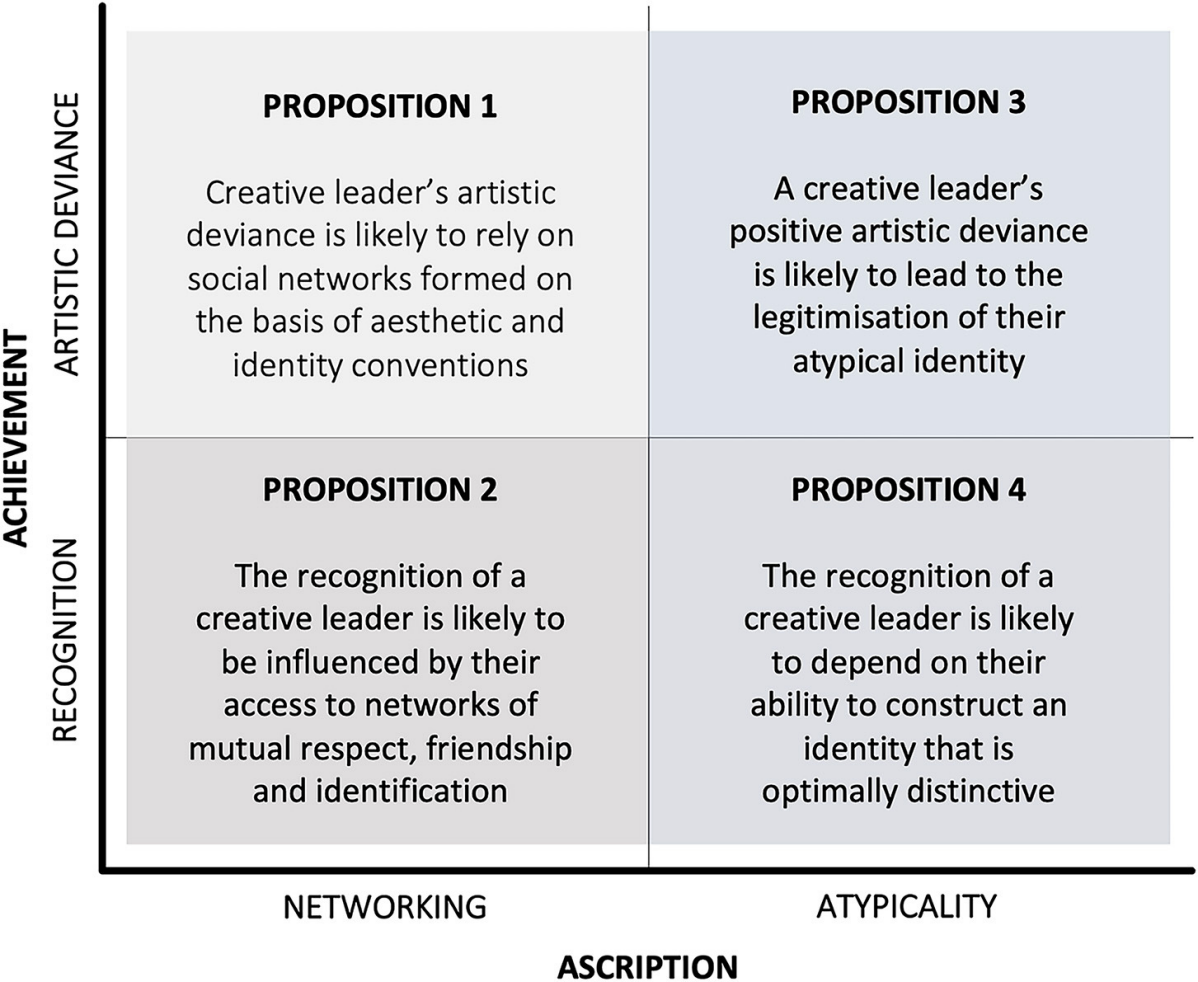
Propositions about achievement and ascription processes of creative leader emergence. Source: The authors.

The emergence of creative leaders relies on both achievement and ascription processes, which are closely interlinked. Our conceptual analysis of achievement and ascription in the art world leads to four propositions (Figure 1). Firstly, art is created as a collective action (Becker, 1982), so that creative outcomes perceived as competent are influenced by social networks formed on the basis of aesthetic and identity conventions. Secondly, the recognition of a creative leader relies on subjective processes of consecration by powerful experts, who are often influenced by the access of a creative leader to networks of mutual respect, friendship and identification (Bourdieu, 1993). Thirdly, a creative leader's positive artistic deviance can also lead to the legitimisation of their identity, including atypical identities (Samdanis and Özbilgin, 2020). Fourthly, the recognition of a creative leader also depends on their ability to engage with identity work and construct an identity that is optimally distinctive (Alvarez et al., 2005): atypical, in terms of differentiating from established conventions; yet culturally relevant, so that they are able to relate and connect with peers, experts and audiences within the art world.

### An Illustrative Case Study of the Emergence of Andy Warhol as a Creative Leader

The achievement and ascription mechanisms of creative leader emergence are illustrated by the case of Andy Warhol (1928–1987), the leading pioneer of the Pop Art movement in New York and creative leader of his art organisation, the Factory (Bockris, 2003). In this paper, we provide an instrumental, qualitative, embedded case study of a creative leader within an art world to illustrate creative leader emergence, based on the processes of achievement and ascription within a historical context (Stake, 1995; Yin, 2017).

We consider achievement and ascription to be socially constructed processes (Berger and Luckmann, 1966; Uhl-Bien et al., 2014; Paunova, 2015), which are based on interpretations and shared meanings produced and reproduced by agents within an art world (Becker, 1982; Bourdieu, 1993; Svejenova, 2018). Although quantitative methods are commonly used in the study of leader emergence (e.g., Judge et al., 2002; Huang et al., 2016; Kwok et al., 2018), the case study method is an established research strategy in the creative leadership literature (e.g., Mumford et al., 2003; Dovey et al., 2017; Abecassis-Moedas and Gilson, 2018; Flocco et al., 2018).

Considering the creative leader as the primary unit of analysis, this paper provides a qualitative historical case study of Andy Warhol and his emergence as a creative leader in the context of the New York art world (Yin, 2017). Our analysis focuses on social processes that enable the construction of the leader instead of leadership, following the distinction made by Paunova (2015, p. 936): “Unlike leader emergence, leadership emergence does not necessarily imply the emergence of a single leader.” Andy Warhol is considered as a leading figure in the Pop Art movement in New York, and he is recognised for his multidisciplinary artistic innovations, and his cultural tactic of constructing an artistic persona to elevate his status in New York's art world (Gopnik, 2020). This is an ideal case with which to test both the achievement and ascription processes of creative leader emergence. We chose to study the emergence of Warhol, because his art studio, the Factory, was a site of social innovation, connectivity and collective creativity which Becker (1982) “would later view as the collective activity of making art worlds” (Hewer et al., 2013, p. 185).

This historical case study is based on secondary data from multiple sources that document the life and work of Andy Warhol, the Factory and the Pop Art period in New York (Table 1). In addition, archival data about the exhibitions of Andy Warhol in New York was sourced online by Leo Castelli Gallery exhibitions archive (https://www.castelligallery.com/exhibitions) and the online archive of the exhibition history of the Museum of Modern Art in New York (https://www.moma.org/calendar/exhibitions/history/).

**Table 1:** The sources of secondary data used in the case study of Andy Warhol.

**References**	**Main focus**	**Type**
**Sources for Andy Warhol**		
Bastian (2001)	The art and life of Andy Warhol	Exhibition catalogue
Bockris (2003)	The art and life of Andy Warhol	Book
Campbell (2015)	Magazine article about Warhol's Polaroids	Magazine article
Crimp (1999)	Contribution of Andy Warhol to Pop Art	Academic article
De Duve (1989)	Contribution of Andy Warhol to Pop Art	Academic article
Gopnik (2020)	The art and life of Andy Warhol	Book
Honnef (2000)	The art of Andy Warhol	Art book
Ketner II, (2013)	The art and identity of Andy Warhol	Art book
Wollen (1997)	The art and life of Andy Warhol	Book chapter
**Sources for the Factory and the New York art world**		
Crane (1987)	The pop art movement in New York	Book
Currid (2008)	The pop art movement in New York	Book
Graw (2009)	Includes information about the factory	Book
Hewer et al. (2013)	The factory as an emergent art world	Academic article
Joseph (2002)	Innovations at exploding plastic inevitable	Academic article
Polsky (2003)	The art market of Andy Warhol	Book
Saltarelli (2018)	A description of Warhol by his contemporaries	Film/Documentary
Schroeder (1997)	The portraits, identity and persona of Warhol	Academic article
Wood (2004)	The pop art movement and the factory	Book
Woronov (1997)	A description of Warhol by his contemporaries	Book chapter

*The themes of achievement and ascription are evident in all sources*.

*Source: The authors*.

Leader emergence is considered to be a within-group phenomenon, while leader effectiveness is seen as a between-group phenomenon (Côté et al., 2010). Within-case analysis is the method used to analyse secondary data, applying our theoretical framework to organise data (Yin, 2017). Data analysis took place by constructing a temporal case narrative, matching evidence about the processes through which Warhol emerged as a creative leader in the art world of New York and the Factory with the mechanisms of achievement and ascription. Our aim in this paper is to illustrate the achievement and ascription processes of creative leader emergence in the art world. The analysis does not aim for generalisations, but for theory development as supported by a force of example (Flyvbjerg, 2006), as the analysis presented here can be conducted for any other creative leaders within the visual arts sector in particular, and the creative industries in general.

#### Andy Warhol Before the Factory

Andy Warhol is known as the leading pioneer of the Pop Art movement in the 1960s in the United States, a movement that originality emerged in the UK in the late 1950s (Bockris, 2003). Born in Pittsburgh in 1928, Warhol moved to New York in 1949 after graduating with a Fine Art degree from Carnegie Institute of Technology. In the 1950s, Warhol launched a successful career in magazine illustration, design and advertising.

The early 1960s was a time of prosperity and euphoria for American society, and a period of growth for the art market of New York (Crane, 1987). Art galleries in New York and Los Angeles introduced Pop Art to American audiences in the 1960s, featuring a new generation of artists, including Andy Warhol and Roy Lichtenstein, together with the former Abstract Expressionists Robert Rauschenberg and Jasper Johns, among others (Gopnik, 2020). The Pop Art movement did not display the features of historical avant-gardes as collectives of artists sharing ideological conventions. It was an artistic idiom that reflected the spirit of the times, using popular images, humour and irony to criticise the complacent consumer society.

Andy Warhol was a creative leader in his art studio, the Factory, in New York between 1964 and 1984 (Gopnik, 2020). The Factory was a place of artistic experimentation, where creativity was approached as a collective and multidisciplinary practise. Warhol was the indisputable leader in his art studio, but his status in the art world had been established in the years before the Factory opened its doors to the underground artists and celebrities of New York who frequented the place. Warhol had his first exhibition as an artist at the Hugo Gallery New York in 1952, at a time when he primarily worked as an illustrator. The blooming of the advertising industry in the 1950s played a significant role in Warhol's career, as the technical expertise, resources and reputation acquired from his work as an illustrator were used later in his artistic production.

In 1959, Warhol created his first Pop Art exhibition, Wild Raspberries, at the Bodley Gallery (Bastian, 2001). He realised early in this career that he had to develop a unique style, which would make him easily recognised in the art world (De Duve, 1989). During a visit to the Leo Castelli Gallery, Warhol saw a Lichtenstein painting of a comic strip, a style that he was also experimenting with at the time (Ketner, 2013). This revelation made him abandon this convention and seek a new style. Warhol was observing conventions introduced by artists such as Robert Rauschenberg and the Greek-born Chryssa, who experimented with transferring ta printed image into canvas (Gopnik, 2020). In the early 1960s, Warhol was searching not only for his artistic style but also for gallery representation.

The following year, 1962, was a turning point in Warhol's career. He discovered the photo-silkscreen technique, a method that became his artistic signature, portraying celebrities such as Marlon Brando, Elvis Presley and Marilyn Monroe (Bastian, 2001). Experimenting with this silkscreen technique, Warhol created art which was aligned with contemporary technical conventions in the art world. In 1962, Warhol exhibited three of his most recognised artworks. Gold Marilyn Monroe was exhibited at the Stable Gallery, and then acquired by MoMA in 1963; the iconic 200 One Dollar Bills was displayed at the Green Gallery, and Campbell's Soup Cans appeared at the Ferus Gallery in Los Angeles (Gopnik, 2020). By the spring of that year, Warhol appeared in the New Talent issue of *Art in America* magazine, one of the most influential contemporary art magazines in the USA (Gopnik, 2020). The New Realists exhibition at the Sidney Janis Gallery was a defining moment for Warhol, as his work was exhibited next to that of his contemporary Roy Lichtenstein and European legends such as Yves Klein (Hess, 2005). In the autumn of 1962, Warhol exhibited his Elvis paintings at Blum Gallery in Los Angeles, where he also visited an exhibition of Marcel Duchamp at the Pasadena Museum of Art. His meeting with Duchamp deeply influenced his conceptual art installations, notably the 1964 Brillo Box sculpture (Ketner, 2013).

From the early 1960s, Warhol made important connexions with art dealers in New York, Los Angeles and Paris, and established friendships with Jasper Johns and Robert Rauschenberg, who he met through the filmmaker and art aficionado Emile de Antonio (Honnef, 2000). There is “good evidence of close ties between Warhol and Rauschenberg. They traded studio visits and Rauschenberg actually went to Warhol for advice on the new art of the photo silkscreen” (Gopnik, 2020, p. 244). Warhol became friends with Henry Geldzahler, curator of contemporary art at the Metropolitan Museum, and “began working with Ivan Karp, director of the Castelli Gallery” (Honnef, 2000, p. 36).

Geldzahler encouraged Warhol to paint the headline 129 Die in Jet! This launched Warhol's Death and Disasters series that also included works including Electric Chair and Race Riot (Bastian, 2001; Ketner, 2013; **Image 1**). In contrast to the light-hearted commodity theme of his previous work, “this strand of artistic experimentation exposed “the dark underside of celebrity, society and wealth, confronting the stark reality of death in America” (Ketner, 2013, p. 33). While Death and Disasters is among Warhol's most powerful works, it was first exhibited in Paris by Ileana Sonnabend in a gallery which was acting almost as Castelli Europe (Gopnik, 2020). The limited commercial interest in this series in New York strained Warhol's relationship with Eleanor Ward of the Stable Gallery, Warhol's main representatives. 1964 found Warhol not only in his new art studio, the Factory, but also under the roof of the Leo Castelli Gallery (Gopnik, 2020).

**Figure 2:**
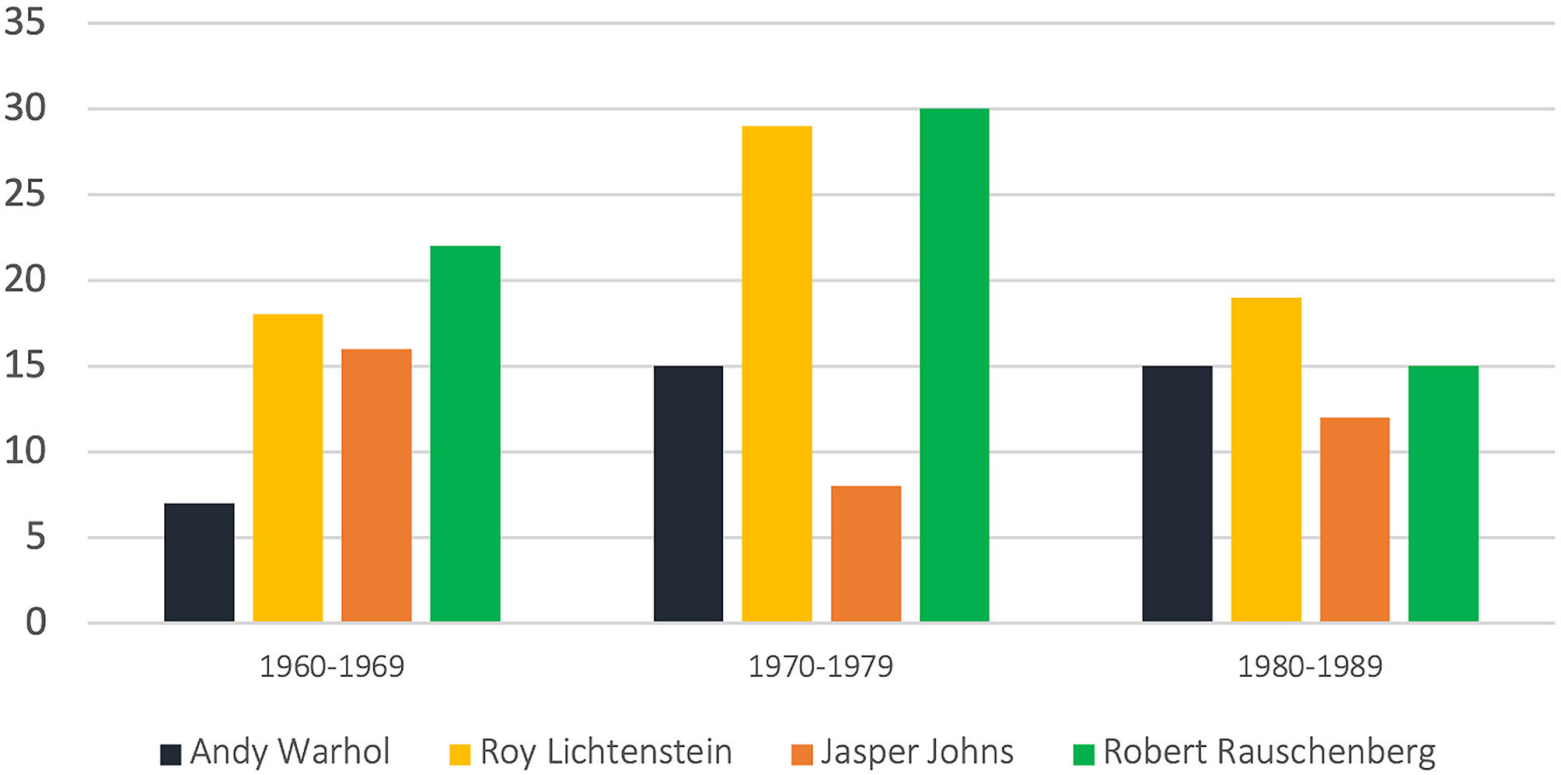
Number of exhibitions at the Leo Castelli Gallery (1960–1989). Source: Developed by the authors based on data from Castelli Gallery online archive.

**Figure 3:**
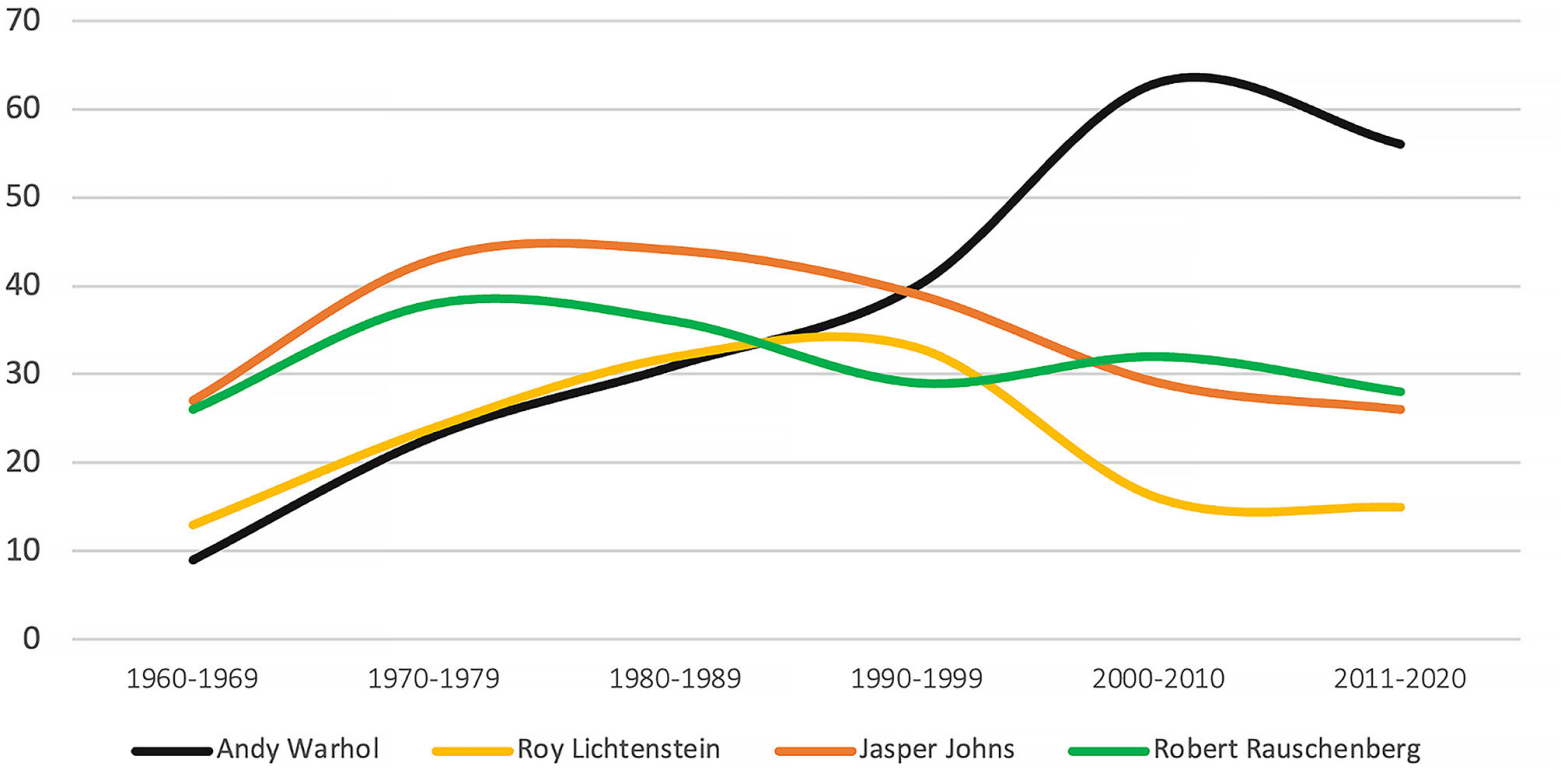
Number of exhibitions (solo and group) at the Museum of Modern Art, New York (1960–2020). Source: Developed by the authors based on data from the Museum of Modern Art online archive.

Castelli was crucial in framing and establishing the Pop Art movement in the 1960s. Compared to his contemporaries in the Pop Art movement, such as Roy Lichtenstein (1923–1997), Jasper Johns (b.1930), and Robert Rauschenberg (1925–2008), Warhol was the least exhibited and recognised artist in the 1960s (Figures 2, 3). Figure 2 shows the number of exhibitions—both solo and group—of these four artists at the Leo Castelli Gallery throughout Warhol's career until 1987, a few years after his death. Of these four artists, Warhol was the least exhibited in the 1960s, and also less exhibited than Roy Lichtenstein in the 1970s and 80s. It is important to note, however, that all four artists, and Warhol and Lichtenstein in particular, contributed to many group exhibitions at the Leo Castelli Gallery, which led to the framing and establishment of the Pop Art movement.

The highest point of consecration for artists is when their work is displayed within—or acquired by—prominent art institutions (Putnam, 2009). Figure 3 shows the number of exhibitions in which each of these four artists participated at the Museum of Modern Art (MoMA), one of the most prominent art institutions for the display of modern art in New York. It should be noted that Pop Art was approached with great scepticism and resistance in the early 1960s by both art critics and art institutions, including MoMA (Gopnik, 2020). Warhol was the least exhibited artist among the four from the 1960s until his death in 1987. Since the mid-1990s, however, Warhol has become the most exhibited Pop artist at MoMA. This has contributed to the establishment of his legacy as the leader of the Pop Art movement (Crimp, 1999).

#### The Rise of Andy Warhol as a Pop Persona

Pop Art was the first art movement in which attention shifted from collective grouping and labelling to the individual, or more accurately, to transforming the image of the individual artist into a brand (Finkelstein, 2007). Andy Warhol underwent a major makeover of his appearance in 1962 and stylized his attitudes to become the epitome of cool: “The essential ingredient of the new Andy Warhol persona was Andy the machine, Andy the android, Andy the asexual creature” (Lee, 2015, p. 29). Warhol's friend and art critic David Bourdon (in Gopnik, 2020, p. 234) noted that Warhol's “metamorphosis into a pop persona” was a calculated and deliberate move.

In the following years, Warhol constructed an atypical image of artist-personality which converged with his artistic oeuvre (Stallabrass, 2006). Geldzahler, a friend and sponsor of his early days claimed that Warhol's appearance and persona influenced the public to identify him with the Pop movement (Honnef, 2000, p. 8):

“Warhol's transformation into a Pop person was thought-out and well-considered. He put his dandified airs behind him when he gradually changed from a worldly-wise person with a subscription to the Metropolitan Opera into a gum-chewing, seemingly naïve teeny-bopper who submitted to the lowest forms of Pop culture.”

The artistic persona that Warhol fabricated was an embodied version of the Pop Art idiom and a real-life manifestation of his artistic intentions (Bockris, 2003). Interestingly, there are two interpretations of Warhol's motive for the creation of his new public identity. On the one hand, the construction of the persona is interpreted as Warhol's attempt to establish himself as a celebrity, or what is known nowadays as a celebrity brand (Preece, 2015). Fashion played a central role in the creation of Warhol as an artist-personality (Wollen, 1997, p. 13):

“Warhol followed English fashion from 1963 when he first met Nicky Haslam, who had come to New York as an art director for Vogue. Magazine art directors were the key figures in Warhol's career, essentially acting as patrons. Gallery owners and directors played a similar role for Warhol in the art world.”

On the other hand, the creation of the artistic persona was a necessity for Warhol due to his social origins, as the son of working-class immigrants, and sexual identity. As Gopnik (2020, p. 477) notes: “being a successful New York artist who was both Pop and gay didn't only put you onstage in the ‘60s, it left you forever in costume.” Within a heteronormative context, Warhol would face similar barriers to those experienced by earlier female artists:

“Beginning already with Georgia O'Keeffe and Frida Kahlo, modern artists who were women had no choice but to build unusual personas for themselves, since Western society didn't offer models for how to be a woman who made art. As a visibly gay man, Warhol found himself pretty much in the same boat as these women” (Gopnik, 2020, p. 473).

Before the creation of the Factory, Andy Warhol had established his status in New York's art world. This status as a celebrity artist was increasing, based on both the new conventions introduced by his art, and his identity that captured the public's imagination as a performance of the spirit of Pop.

#### The Emergence of Andy Warhol as a Creative Leader at the Factory

In 1964, Warhol created his famous studio the *Factory*, an art organisation and collective that included his personal assistants, creative associates, visual and performing artists, stylists, musicians, actors, filmmakers and other celebrities (Bockris, 2003). Warhol and the Factory played a leading role in the production of Pop Art by creating “a social cycle whose members were linked to one another through indirect as well as direct ties, which permitted information and ideas to spread through the entire group” (Crane, 1987, p. 30). Warhol was perceived in the art world as a magnet and an individual with extraordinary charisma (Mead, in Schoor, 2008), who had the ability “to select and elect the chosen ones” (Hewer et al., 2013, p. 188). The art historian Klaus Honnef (2000, p. 72) describes Warhol's relationship with his followers: “Warhol fascinated and stimulated this group of strange characters and they in turn served him as recipients and mediums of something which, for lack of better term, can be defined as the spirit of the times.”

The Factory can be described as a directing creative context, since “nothing went out of the studio door that had not received the master's explicit seal of approval” (Honnef, 2000, p. 72). Warhol would select themes, images and colours. His associates such as Gerard Malanga would then produce the silkscreen printings for his final approval. If openness to experimentation was one of Warhol's key personality traits, his spirit of research and his unique gaze on people, places and things were most frequently mentioned as his main creative skills (Gopnik, 2020). Warhol is often portrayed as always carrying his Polaroid: whichever social occasion he attended, “he'd record it—and the people he met—for his ‘visual diary'… Out would come the Polaroid which accompanied him everywhere Snap” (Campbell, 2015). Many of these Polaroids were used in silkscreens, especially to make portraits.

The Factory can also be described as an integrative creative context. During the Factory years, Warhol encapsulated in his work the collective nature of creativity, as art, fashion, music, film, performance, and design were constantly engaging each other, facilitating the sharing of ideas and resources across creative sectors (Currid, 2008, p. 15). The Factory was an open social space in the underground scene, which attracted different kinds of audiences and followers: ambitious but resourceless emerging artists; marginalised figures, transvestites and gay people, some of whom would become Warhol superstars, emerging celebrities like Edie Sedgwick, and established artists, actors, writers, musicians such as Jack Kerouac, Allen Ginsberg, and Dennis Hopper, Barnett Newman, Judy Garland, and the Rolling Stones (Bockris, 2003).

Warhol “had been trained as a commercial artist,” but during the Factory years “had created a completely new type of artist, which irritated, shocked and changed the world of art” (Honnef, 2000, p. 7). The Factory was a “place of social inclusion, of play, performativity and the feminine deflation of heroic male imposture [which] distanced Warhol's project from the aggressive masculine topos of the Abstract Expressionists” (Wood, 2004, p. 180). The new conventions at the Factory that shocked the art world were not just aesthetic, but also transformed into atypical identities, and the bohemian and transgressive lifestyles shared by this artistic milieu: “The Factory of the early 1960s can be understood as a machine that not only staged transgressive ways of life, but also profited from people's willingness to perform them” (Graw, 2009, p. 174).

Although Warhol's involvement with film started before the Factory, this creative context was an ideal space for documenting New York's underground scene. Films such as Chelsea Girls (1966), created by Warhol and his associate Paul Morrisey, were spontaneous documentations of happenings at the Factory (Bockris, 2003), which was the social and creative context in which “different concepts of sexual identity” were captured by Warhol's films (Graw, 2009, p. 174). In 1964, Warhol won a Film Culture award at the New York Film Festival for his short films Eat, Sleep, Kiss and Haircut, but film critics sidelined his films as avant-garde art or Pop films which would barely reach the mainstream. Warhol acted as producer of his films and referred to his filmmaking activities as “a ‘frightfully expensive' hobby that brought in no cash to speak of: ‘All my painting money goes into it'” (Gopnik, 2020, p. 416).

Warhol also acted as manager for the rock music band The Velvet Underground and Nico, which acted as a house band at the Factory in the mid-1960s (Gopnik, 2020). The German singer, model, actress and superstar Nico was introduced as a vocalist at Warhol's suggestion. Warhol also played a major role in the production of the band's first album in 1967, and designed their legendary banana album cover. In 1966 and 1967, Warhol organised a multimedia series of events called Exploding Plastic Inevitable (E.P.I.). The media theorist Marshall McLuhan (1967, in Joseph, 2002) described E.P.I. as an original, multidirectional, synaesthetic, and interactive audiovisual experience. E.P.I. included a projection of Warhol films, live music by The Velvet Underground and Nico, dance by Gerard Malanga and superstars Mary Woronov and Ingrid Superstar, and the innovative lighting design of Danny Williams (Woronov, 1997; Joseph, 2002; Hewer et al., 2013; Gopnik, 2020).

Despite the artistic novelty generated at the Factory, its cultural production relied on precarious creative labour (McRobbie, 2016; Samdanis and Lee, 2019). Graw (2009, p. 174) claims that “there was in fact a great deal of literal exploitation in Warhol's film production: The actors were not paid. One might argue that the Factory profited from a willingness to work without pay that grew in direct proportion to the symbolic capital and fame that could expected in return.” However, members of the Factory hold different views. For instance, in a recent documentary about the life of Warhol (Saltarelli, 2018), the superstar Cherry Vanilla explained that, although they were paid little or nothing at the Factory, emerging performing artists in the 1960s otherwise had very limited access to resources and opportunities to perform, which is what Warhol offered to them. Gopnik (2020) portrays Warhol as caring for his superstars, attempting—where possible—to provide advice and guidance. This is in line with the claim of Bockris (in Hewer et al., 2013, p. 191) that Lou Reed, the singer of The Velvet Underground, is “a very good example of someone whose life was changed just like that after meeting Andy.”

Although Warhol's identity and lifestyle energised the Factory's artistic milieu, they made it more difficult for him to reach the higher levels of institutional validation. Henry Geldzahler excluded Warhol from the roster of artists who would represent the USA at the 1966 Venice Biennale, on the basis that including him would put Geldzahler's status at the Metropolitan Museum of Art at risk: “The trustees were very edgy about me,” Geldzahler said (Gopnik, 2020, p. 513), adding that “Andy then had the Velvet Underground and was starting the film thing.” While Geldzahler was allegedly looking for post-Pop art, he included Roy Lichtenstein, “Warhol's rival as King of Pop” (Gopnik, 2020, p. 513). According to Gopnik (2020, p. 513):

“The real problem with Warhol seems to have been less with his Pop Art *per se* than his increasingly unserious pop-cultural profile. Geldzahler's more conservative model of art would include a ‘straight' Pop painter like Lichtenstein but couldn't expand to include a Pop—or pop—figure like Warhol.”

The Factory enabled Warhol to be both an insider and outsider in the art world, as he maintained autonomy while forming strong connexions with the prominent art dealers, creatives and celebrities of the time. In 1968, an unexpected incident changed his life forever. Valerie Solanas, a member of the Factory, attempted to assassinate Warhol, claiming that he had too much control of her life (Harding, 2012). Although Warhol was initially pronounced clinically dead, he survived the attack, leaving hospital a month later. After this near-death experience, Warhol re-emerged in the persona of the CEO of Andy Warhol Enterprises Inc., becoming more entrepreneurial as portraitist, publisher, celebrity and salesman (Gopnik, 2020).

In 1969, Warhol launched *Interview* magazine, which became “an instrument to connect with elite celebrities like Yoko Ono, John Lennon, Mick and Bianca Jagger,” and to source portrait work and revenue for the Factory (Gopnik, 2020, p. 766). Warhol was often criticised as being too commercial, a criticism he deflected with irony or apathy (Schroeder, 1997; Graw, 2009). In fact, he launched a new era for the art world, an era in which the art and business are intertwined. After 1968, Warhol started using popular patrons, such as Mick Jagger, Liza Minnelli, John Lennon and Mao Zedong, which also indicates a shift toward more mainstream themes, contributing to Warhol's commercial success in the art market (Polsky, 2003). In his book The Philosophy of Andy Warhol, Warhol (1975, in Bastian, 2001) claimed that “making money is art and working is art and good business is the best art.” During the 1980s, Warhol enjoyed the status of art celebrity, being recognised as a pioneer of Pop Art while also collaborating with the new generation of avant-garde artists, such as Jean-Michel Basquiat (Bockris, 2003). He died in New York at the age of 59 from cardiac arrhythmia. Warhol was a multidisciplinary artist, a celebrity, businessman and performer who “fused high art, low culture, high society and the avant-garde into a distinctive amalgamation that attracted the attention of millions and influenced generations of artists” (Ketner, 2013, p. 7).

## Discussion

This paper addresses a theoretical gap in the creative leadership literature: how do creative leaders emerge within social networks? The creative leader is the primary source of creative thinking and behaviour within a creative context, such as an art world (Becker, 1982; Mainemelis et al., 2015). The literature on creative leadership outlines the key personality traits of creative leaders, such as being open-minded, open to new experiences, imaginative, intelligent, curious and resourceful (Epitropaki et al., 2018); the key skills, such as creative thinking and problem-solving (McClean et al., 2018); the key states, such as creative self-efficacy (Huang et al., 2016); and the key behaviours which are contingent on the creative contexts of facilitating, directing and integrating the creative contributions of leaders and followers (Mainemelis et al., 2015; Mainemelis, 2018).

**Figure 4:**
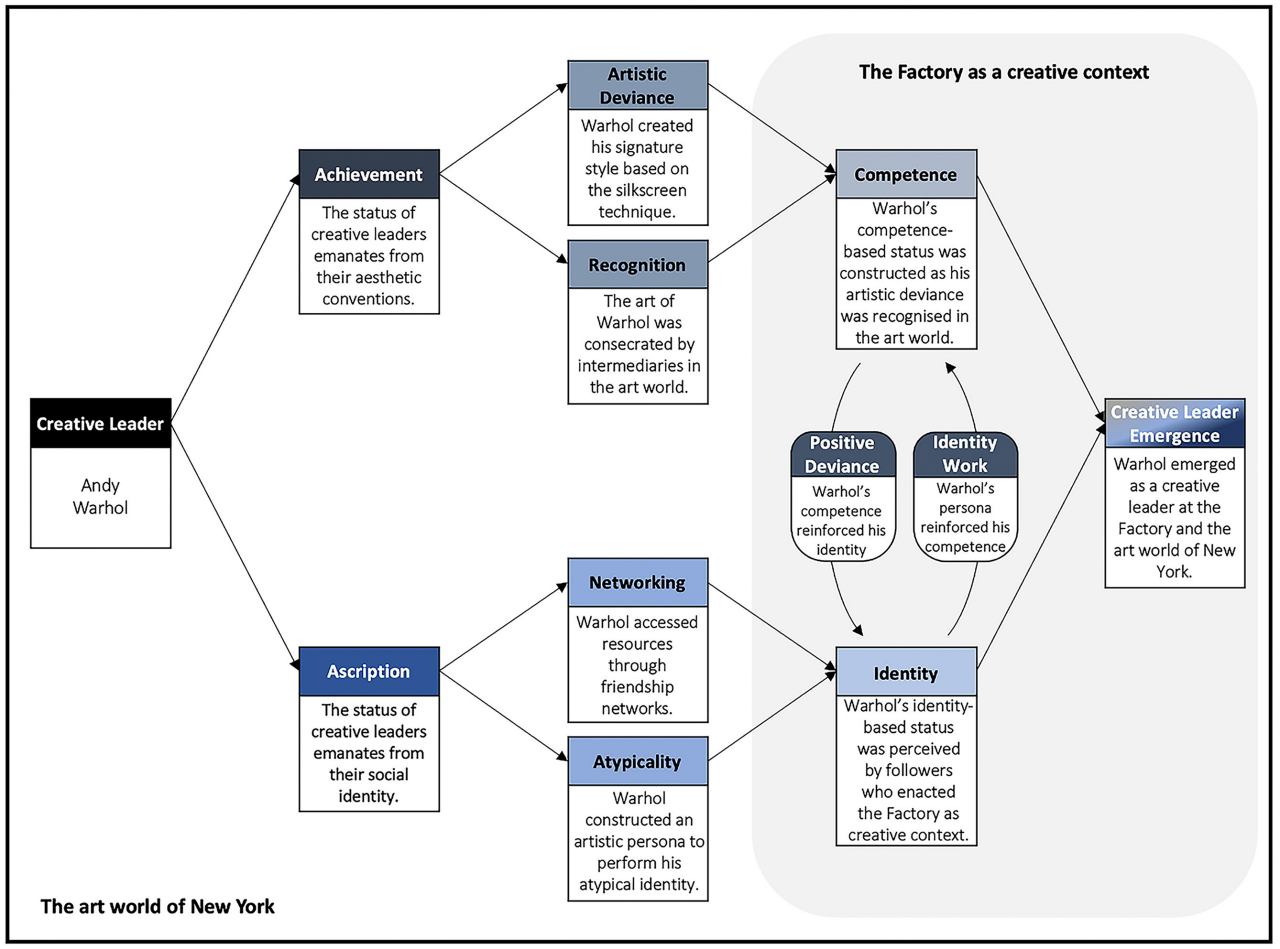
The emergence of a creative leader within the art world illustrated by the case of Andy Warhol. Source: The authors.

Although many of these traits, states and behaviours are typical of creative leaders, very few creative leaders have influenced a creative context or art world as much as Andy Warhol did in the art world of New York and the Pop Art movement. Pop Art in the USA would never be the same without Andy Warhol, but the question arises: did Warhol emerge as a creative leader only because of his charisma or genius? Asserting this would result in missing important lessons about the ways in which creative leaders emerge within an art world. In this paper, we do not attempt to explain the emergence of creative leaders based on the causal relationship between traits, states, behaviours and leader emergence. Instead, we follow a relational view (Uhl-Bien et al., 2014), according to which the emergence of a creative leader results from context-specific interactions and relationships between leaders and followers. In the art world, these interactions and relationships are shaped by conventions, which are similar to perceptions of aesthetics and taste that are shared by creative leaders and others (e.g., assistants, peers, experts, and audience). Based on this relational view, we argue that creative leaders emerge once their interactions and relationships with others in the art world result in the elevation of their status. Figure 4 depicts our conceptual analysis of creative leader emergence, and way in which this framework is exemplified by Andy Warhol.

The contemporary art world is a challenging context as the status, and therefore the emergence, of creative leaders both depend on the acceptance of new conventions by others (Stamkou et al., 2018). Becker (1982) suggests that artists innovate or introduce new conventions when the demand for current conventions declines in the art world. Although this process points out the motivation of artists when they engage with artistic innovation, it does not explain how artists emerge as creative leaders by introducing new conventions. Stamkou et al. (2018) argue that new conventions are introduced based on processes similar to leader emergence, as described by Hollander's (1958) idiosyncrasy credit theory. Leaders initially build their status by producing conventional work to prove their competence to in-group followers. Once established, they use their credit to produce novel work that deviates from conventions. This process explains how leaders emerge and new conventions are introduced, as leaders become prototypical within a group by proving their competence and differentiation to followers.

The idiosyncrasy credit theory can partly explain how Warhol emerged as a creative leader. He deviated from existing conventions at the beginning of his career, when he used the comic strip style featured in the work of Roy Lichtenstein, but engaged with emerging conventions in printmaking, alongside Chryssa and Robert Rauschenberg, which resulted in the pioneering of his own silkscreen printing technique. This technique enabled him to produce his first iconic portraits of Marlon Brando, Elvis Presley, and Marilyn Monroe. Therefore, the idiosyncrasy credit theory is partly applicable to Warhol, as he first established his status in the art world in the early 1960s as a Pop artist, and then deviated in his Factory period by introducing new conventions based on his collective multidisciplinary work. However, the idiosyncrasy credit theory cannot fully explain the emergence of creative leaders from social networks, because the construction of their status within social networks is not necessarily based on leader prototypicality. Instead, their competence and status are often determined by peers, experts and audiences that share their conventions within the art world (Becker, 1982).

Warhol acquired his status as a leading Pop artist in the art world, when his work was featured in prominent galleries and art institutions. This suggests that creative leader emergence in the visual arts relies not only on the perceptions of followers within a group, but also on formal organisations and institutions, as well as informal networks (Bourdieu, 1993; Patriotta and Hirsch, 2016). The achievement mechanism is implicitly based on the idea that a leader emerges when the majority of followers perceive their competences and functional behaviours to be leader-like (Mainemelis et al., 2015; Paunova, 2015). However, this view dismisses the role of powerful intermediaries in consecrating new conventions as legitimately distinctive (Bourdieu, 1993). In the visual arts, the creative context shifts from the organisation to the art world. As a result, we argue that creative leader emergence and creative behaviour are contingent not only on organisational creative contexts (Mainemelis, 2018), but also on the power structures and selection mechanisms that exist within an art world. More broadly, it is implicit in the leader emergence literature (Paunova, 2015; Acton et al., 2019), and also in Becker (1982), that leaders and artists receive recognition based on a consensus within groups or the art world, respectively. However, this view will be limited if the impact of power structures on leader emergence is not taken into account.

Following Paunova (2015), we developed a second mechanism of creative leader emergence that emphasises ascription. The rationale for this mechanism is that the nominal characteristics and social identities of creative leaders can also determine their status and emergence within an art world. Becker's (1982) concept of conventions refers mainly to the aesthetic conventions of an artwork. An extended version of conventions can also include the identities, vocabulary, storeys, styles, and lifestyles shared between leaders and followers, which enact connexions, relationships, and communities within an art world (Cluley, 2012).

The identity of a creative leader can be a source of recognition and status construction in the art world. This is aligned with Mainemelis et al. (2015, p. 434–35) who focus on the context of haute cuisine and state that “becoming a top chef” is “not a question of being creative in mixing ingredients and crafting recipes, but a question of doing so in a way that leads up to the formation of an authentic identity which challenges or/and replaces ideas and practises in the field.” The formation and communication of authentic identities by creative leaders are ways of branding their authenticity (Preece, 2015), signalling their conventions, and attracting the kind of followers who identify with them. The status that emanates from the identity of a creative leader can therefore attract followers and generate cooperative links in the art world (Becker, 1982). Such a generative dimension of creativity within social networks (Cattani et al., 2015) is described by Hewer et al. (2013) as “art worlding,” denoting the enactment of an art world based on new cooperative links.

Warhol notoriously transformed his appearance and changed his public attitude, manifesting and performing an atypical identity through his artistic persona. The term atypical seems more appropriate than authentic to describe an artist who literally embraced reproduction in his work, in terms of using ready-made objects and images in his artistic production. Authenticity in business contexts has received attention, as Sparrowe (2005, p. 420) states: “In Authentic Leadership (2003, p. 11), Bill George argues that ‘being yourself; being the person you were created to be' rather than ‘developing the image or a persona of a leader' is the way to restore confidence in business organisations after Enron and Arthur Andersen.” However, it would have been problematic for Warhol, a queer man, to be authentic in a heteronormative context such as the USA in the 1960s (Gopnik, 2020). Warhol's status and identity played a vital role in assembling the milieu at the Factory. He used his friendship networks and connexions to source key associates for his creative projects, while many of his associates, including his superstars, expected symbolic returns, such as fame by participating in his projects for little or no pay (Graw, 2009).

The case of Warhol demonstrates that achievement and ascription processes are not mutually exclusive mechanisms, but interact with and reinforce each other. Warhol's artistic oeuvre cannot be separated from his atypical identity as performed through his artistic persona. The recognition of a creative leader's positive artistic deviance can also lead to the legitimisation of their atypical identity. In the case of Warhol, the recognition of his artistic deviance by an influential part of New York's art world also led to the legitimisation of his persona identity.

Through his persona, Warhol was distinctive yet popular, being both an insider and an outsider to the New York's art world. As an insider to the art world, Andy Warhol networked with powerful art dealers, and formed relationships with art professionals and creatives, which influenced his recognition and achievement. However, Warhol maintained his capacity to innovate for more than two decades by also being an outsider, as the work produced within the Factory was based on the collective action of an artistic milieu that shared both aesthetic and identity conventions. His persona identity allowed Warhol to perform a balancing act between the mainstream and the avant-garde. This balancing act, which of course was not lacking in criticism, allowed him to attract further followers and audiences, and build his fame as a pioneer of Pop Art in New York. By engaging with identity work, Warhol maintained his artistic deviance over time, a point that demonstrates that the achievement of a creative leader cannot be distinguished from their identity.

## Conclusion

This conceptual paper addresses the ways in which creative leaders emerge within social networks, a topic which has previously received limited attention in the literature on creative leadership. Based on a relational perspective on creative leader emergence (Uhl-Bien et al., 2014), we have blended the literature on sociology of art (Becker, 1982) and leader emergence (Paunova, 2015), to introduce two processes for creative leader emergence in the context of the art world.

As illustrated by the historical case of Andy Warhol, achievement shows creative leader emergence to be a status-construction process through which the conventions of a creative leader are recognised by peers, intermediaries and audiences, while ascription explains creative leader emergence as a process of status construction in which followers endorse the identity of a creative leader. When the achievement and ascription processes are combined, creative leader emergence is likely to introduce innovations in the form of new conventions, and enact structures, as new cooperative links are formed. Our conceptual analysis leads to two theoretical implications. Firstly, competence and identity reinforce each other in the process of creative leader emergence. The recognition of a creative leader's artistic deviance within an art world can result in the legitimisation of their atypical identity. Secondly, through identity work, creative leaders can be both insiders and outsiders in the art world, maintaining their artistic deviance while accessing resources from the core of the art world.

This conceptual paper also contributes to the wider literature on leader emergence. More specifically, we complement the existing literature that scrutinises leader emergence within groups (e.g., DeRue et al., 2015) and leaderless groups (e.g., Ensari et al., 2011), based on mechanisms of leader prototypicality, by demonstrating that leaders can also emerge while they enact groups, networks and communities of followers based on mechanisms of atypicality. While leader prototypicality explains leader emergence, as followers perceive the leader as being one of them (Steffens et al., 2013), leader atypicality explains leader emergence as followers are inspired by the legitimately distinctive identity of a leader. However, leader prototypicality and atypicality are not either/or phenomena. While optimal distinctiveness is a deliberate strategy or cultural tactic, according to which leaders aim for both assimilation and differentiation from others (Brewer, 1991; Alvarez et al., 2005), leader atypicality is a leader emergence mechanism through which followers endorse the atypical identity of a leader. However, an atypical creative leader, like Warhol, can engage with identity work and aim for optimal distinctiveness.

This paper is subject to limitations which could be addressed in future research. Firstly, prior research explores the relationship between the role identity of leaders and leader emergence (Kwok et al., 2018). As Kwok et al. (2018) suggest, networks of friendship can affect leader emergence, a point which we have incorporated into our conceptual development. However, it is not certain whether creative leaders within social networks develop a leader role identity (Kwok et al., 2018), or a motivation to lead (Randel and Jaussi, 2019), as may be the case for creative leaders within existing groups and formal organisations. Thus, empirical research focusing on the identities of creative leaders and their relationships with their followers is required to understand whether creative leaders develop the role identity of a leader, and how this identity is integrated with other social identities.

Secondly, based on the ascription mechanism of creative leader emergence, future research could more explicitly analyse the role of social identities, such as gender, social class, race, religion and intersectional identities in creative leader emergence and effectiveness (Rosette et al., 2016). Andy Warhol, through the creation of his artistic persona, managed to overcome some of the social barriers associated with his queer identity (Gopnik, 2020). As these barriers are associated with the 1960s American context, so more research is required to understand the barriers and enablers for atypical creative leaders in more recent and current creative contexts.

Thirdly, in this paper we have used the emergence of Andy Warhol as a creative leader as a case study to illustrate our conceptual analysis. As part of this, we also mentioned an important incident later in his career and life—the assassination attempt by Valerie Solanas in 1968—while stressing that Warhol then re-emerged as a more corporate leader. Future research could focus on the process of inside-out transformation following psychological and physical trauma, examining how they affect leader emergence, development and efficiency. Finally, in the context of increasing digitalisation of creative industries (Khaire, 2015), it will be interesting to investigate collective and distributed modes of creative leader emergence in sectors such as art, architecture and fashion, which have changed significantly in recent decades due to digital transformation.

## Author Contributions

All authors listed have made a substantial, direct and intellectual contribution to the work, and approved it for publication.

## Conflict of Interest

The authors declare that the research was conducted in the absence of any commercial or financial relationships that could be construed as a potential conflict of interest.

## Footnotes

^1^Implicit theories of leadership and creativity focus on mental representations of leaders and creative individuals (i.e., how leader and creatives *should* be) in the minds of others (Epitropaki et al., 2018).
